# Enhancing stability of essential oils by microencapsulation for preservation of button mushroom during postharvest

**DOI:** 10.1002/fsn3.129

**Published:** 2014-06-01

**Authors:** Majid Alikhani-Koupaei, Meisam Mazlumzadeh, Mohamadmehdi Sharifani, Mohamad Adibian

**Affiliations:** 1 Higher Educational Complex of SaravanSaravan, Iran; 2 Faculty of Plant Production, Horticulture Department, Gorgan University of Agricultural SciencesGorgan, Iran

**Keywords:** *Agaricus bisporus*, rosemary, thyme

## Abstract

Fresh button mushrooms (*Agaricus bisporus* L.) are sensitive to browning, water loss, and microbial attack. The short shelf-life of mushrooms is an impediment to the distribution and marketing of the fresh product. Essential oils outstand as an alternative to chemical preservatives and their use in foods meets the demands of consumers for natural products. To resolve controlled release of oil and increase in antioxidant and antimicrobial activities, the oil was incorporated into microcapsules. Effects of microcapsulated thyme (*Thymus vulgaris* L.) and rosemary (*Rosmarinus officinalis* L.) on quality of fresh button mushroom were compared. Physicochemical qualities were evaluated during 15 days of storage at 4 ± 0.5°C. All treatments prevented product weight loss and decrease in polyphenoloxidase and peroxidase activities during storage. Color and firmness, microbiological analysis, and total phenolic content caused the least change. With use of microencapsulated oils, mushrooms were within acceptable limits during 10 days of storage. Microencapsulated rosemary oil produced the highest beneficial effects and has potential to improve quality of button mushrooms and extend shelf-life.

## Introduction

The white button mushroom (*Agaricus bisporus* L.) has beneficial nutritional, organoleptic, and medicinal properties (Beelman et al. 2003). However, button mushroom has a shelf-life of only 3–4 days due to browning, water loss, senescence, and microbial attack (Beelman et al. 2003). Mushrooms are conventionally packed in plastic trays covered with perforated polyvinyl chloride film and refrigerated (Jiang 2013; Gao et al. 2014). High humidity, created by a high-transpiration rate and poor water vapor permeability of the film, causes condensation inside the package. The short shelf-life of fresh mushroom is an impediment to distribution and marketing. Prolonging postharvest storage, while preserving quality, would benefit the mushroom industry and consumers (Kim et al. 2006; Lagnika et al. 2011). Many essential oils and their constituents have antimicrobial activities, rendering them alternatives to synthetic fungicides (Adam et al. 1998). Essential oils are generally regarded as safe and should be acceptable to consumers. The multicomponent nature of essential oils makes it difficult for pathogens to build up resistance. Application of essential oils as alternatives, or in addition to synthetic fungicides, can prolong the useful shelf-life in postharvest (Alikhani et al. 2009; Ayala-Zavala et al. 2009).

Many essential oils are instable (Hsieh et al. 2006). Microencapsulation may increase stability and modify release characteristics. Microencapsulation is not a product, or a component of a product. It is a process of enclosing micron-sized particles of solids, or droplets of liquids or gases, in an inert shell, which in turn isolates and protects them from the external environment (Wieland-Berghausen et al. 2002; Jaya et al. 2010). The inertness is related to the reactivity of the shell with the core material. This technology is mainly used for, purpose of protection, controlled release, and compatibility of core materials (Hsieh et al. 2006). The product of the microencapsulation process is a “microcapsule” of micrometer size (>1 *μ*m), having a spherical or irregular shape. Microcapsules can be divided into two parts, the core and the shell (Polk et al. 1994; Wieland-Berghausen et al. 2002). The core contains the active ingredient; the shell protects the core from the external atmosphere (Bungenberg de Jong and Kruyt 1949; Polk et al. 1994).

Rosemary (*Rosmarinus officinalis* L.) and thyme (*Thymus vulgaris* L.) oil microencapsulation can be prepared by a coacervation method using chitosan and *β*-cyclodextrin as coating materials (Bhandari et al. 1998; Xing et al. 2011).

Chitosan is a modified natural carbohydrate polymer derived from chitin which has is found in a wide range of natural sources such as crustaceans, fungi, insects, and some algae, and is a coating material to prolong shelf-life (Chien et al. 2007).

The study was undertaken to determine the efficacy of antimicrobial and antioxidant activity of microencapsulated rosemary and thyme oils on mushroom tissue browning and quality characteristics after cold storage.

## Materials and Methods

### Essential oil extraction

Dry plant materials were distilled for 24 h in a steam distiller with an aqueous phase recycling system, using a plant material:water ratio of 2:1. The distillation time was about 2 h. The oil obtained was separated from the aqueous solution and dried by treating with anhydrous Na_2_SO_4_. Each essential oil was transferred into a dark glass flask filled to the top and kept at a temperature of 4°C until used.

### Preparation of essential oils microencapsulation

The microencapsulation process was by coacervation coupled with vacuum-drying (Bhandari et al. 1998; Ojagh et al. 2010;) with minor modification. An aqueous dispersion containing *β*-cyclodextrin (5 g) was dissolved in 200 mL of distilled water at 70°C on a hot plate. After cooling to 40°C, 0.5-mL essential oil in ethanol (1:1 v/v) was slowly added to the solution with continuous agitation, to give a molar ratio of essential oil/*β*-CD of 0.4–2.4. The vessel was stirred for 3 h, and chitosan (1.0%), essential oil (0.15%), ethanol (20%) Tween 80 (0.2%), and glycerol (0.75%) were added. The pH was adjusted to eight by addition of a suitable amount of NaOH 1 N and the complex solution stirred by a magnetic stirrer at room temperature for 1 h. The hardened microparticles were filtered, rinsed with cold water, and dried at 30°C for 48 h under vacuum.

### Treatment

Button mushrooms sporophores from the local market of Esfahan city were selected for uniform size, maturity, and absence of mechanical damage. Five *μ*L·L^−1^ each of rosemary and thyme oils (RO and TO) (Gao et al. 2014) and 0.3 g of microencapsulated rosemary and thyme oils (MRO and MTO) (Xing et al. 2011) were used. Button mushrooms were placed in polyethylene (PE) film packaging, with filter paper inside the cover. A total of 10 *μ*L of each essential oil was spotted onto the filter paper. Microencapsulated oils were weighed and sealed in the synthetic package (4 × 5 cm), in which two small holes were made with a needle before placing in the PE packaging. A total of 60 button mushrooms for each treatment were used and PE packaging stored at 4 ± 0.5°C in a refrigerator and three replicates from each treatment group were taken at 5 day intervals for up to 15 days of storage.

### Quality evaluation

Firmness of mushrooms (N) was with a TA-XT2 penetrometer (Stable Microsystems Texture Technologies Inc., Godalming, Surrey, UK) by measuring force required for a plunger 2 mm in diameter to penetrate 5 mm into the cap surface of mushroom at a rate of 2 mm·sec^−1^.

Weight loss was detected by the differences between initial weight and final weight divided by the initial weight.

Color on opposite sides of mushroom caps was measured with a Minolta CR-300 colorimeter (Minolta Co. Ltd., Tokyo, Japan) in the Commission Internationale de l'Eclairage (CIE) L*a*b* mode CIELAB color space. The parameters determined where *L* (*L* = 0 [black] and *L* = 100 [white]), *a* (−*a* = greenness and +*a* = redness), *b* (−*b* = blueness and +b = yellowness), and compared to ideal mushroom color values of L* = 97, a* = −2, and b* = 0 using Δ*E* as described by the equation (Gao et al. 2014): Δ*E* = [(*L* – 97)^2^ + (*a* − (−2))^2^ + *b*^2^]^1/2^; where Δ*E* indicates degree of overall color change compared to color values of an ideal mushroom. The browning index (BI), which represents purity of brown color (Palou et al. 1999), were calculated according to the equation: BI = [100 (*x* − 0.31)]/0.172, where *x* = (*a* + 1.75*L*)/(5.645*L* + *a* − 3.012*b*).

Percent open caps were determined from a known number of mushrooms. The criteria for judging percent open caps were based on development of the umbrella-like shape of the cap and detachment of the veil.

The marketability of mushrooms was with a hedonic 5-point scale for color, texture, and percent open caps by a trained panel, scoring 1 (lowest) to 5 (highest), according to Gao et al. (2014).

### Microbiological analysis

Microbiological characteristics of a 10-g sample were obtained after homogenization in 90-mL 0.1% peptone water (0118-17-0; Difco, Hanley Industrial Ct, St. Louis, MO). Other dilutions were prepared from a 10^−1^ stock solution. Total count was determined using the pour plate method and Plate Count Agar (0479-17; Difco) as the medium. Plates were incubated at 35°C for 48 h (Harrigan 1998). Three samples in each group were analyzed.

### Total phenolic determination

Total free phenolic content was estimated spectrophotometrically, using the method of Singleton and Rossi (1965). Briefly, mushrooms (5 g) were pureed and homogenized with 25-mL water. The mixture was centrifuged at 10,000*g* for 15 min at 4°C. Four mL of sodium carbonate solution (75 g·L^−1^) was added to 1 mL of centrifugate, then the reaction mixture was reacted with 5 mL Folin–Ciocalteu solution (0.2 N). Absorbance of the solution at 765 nm was measured after 2 h (model 1601; Shimadzu, Tokyo, Japan). The standard curve was established using gallic acid.

### Determination of ascorbic acid

The 2,6-dichloroindophenol titrimetric method (Helrich 1990) was employed to determine ascorbic acid content of mushroom. Tissues (10 g) were homogenized with 10 mL of 3% (v/v) metaphosphoric acid in a grinder. The extract was made up to a volume of 100 mL and then centrifuged (model Sorvall RC-5C; Du-Pont, Wilmington, DE) at 5000*g* for 15 min at 25°C. The supernatant phase was collected and analyzed for the amount of ascorbic acid as determined by titration with 2,6-dichlorophenolindorhenol which had already been standardized against standard ascorbic acid.

### Activities of polyphenoloxidase and peroxidase enzymes

For analysis of enzymatic activities, mushroom tissues (4.0 g) were homogenized with 12 mL of 50 mmol/L K-phosphate buffer (pH 7.3), containing 1 mmol/L ethylenediaminetetraacetic acid and 2 mmol/L dithiothreitol. After centrifugation at 10,000*g* for 15 min at 4°C, the supernatant was collected and used as the crude enzyme extract for the polyphenoloxidase (PPO) and peroxidase (POD) assays. The protein content was determined according to the method of Bradford (1976), with bovine serum albumin used as the standard.

PPO activity was measured by incubating 0.5 mL of enzyme extract in 2.5 mL of buffered substrate (100 mmol/L sodium phosphate, pH 6.4 and 50 mmol/L catechol), and the change monitored at 398 nm absorbance (Wang et al. 2004). One unit of activity of PPO was defined as the amount of enzyme to cause 0.01 absorbance increase per minute.

POD activity was measured spectrophotometrically, using the substrate guaiacol (Moerschbacher et al. 1988). The reaction mixture for determination of POD activity consisted of 50 mmol/L sodium phosphate buffer (pH 6.0), 5 mmol/L guaiacol, 5 mmol/L H_2_O_2_, and 50 *μ*L of tissue extract. One unit of POD activity was defined as the amount of enzyme that caused a change in absorbance at 470 nm of 0.01·min^−1^.

### Statistical analysis

The research was established according to complete randomized design (CRD) with three replicates. All data obtained from the trial were analyzed using analysis of variance (ANOVA) and using the computer software SPSS version 15.0 (SPSS Inc., Woking, Surrey, UK). Means were compared using the least significant difference test at the *P* < 0.05.

## Results and Discussion

### Firmness

Firmness reflects changes in metabolic and water content. Firmness at harvest and during storage was reduced (Table 1). The reduced firmness was lowest in those treated with microcapsulated thymus and rosemary oils. The greatest reduction in firmness was for the controls. Softening can occur due to degradation of cell walls in postharvest by bacterial enzymes and increased activity of endogenous autolysins (Zivanovic et al. 2000). Essential oil by antioxidant phenolics could reduce the action of cell-wall degrading enzymes (Barreit and Gonzalez 1994).

**Table 1:** ANOVA of unencapsulated and microencapsulated thyme and rosemary oils on firmness, weight loss, percent open caps, marketability, total phenolics, ascorbic acid, total aerobic plate count of microorganisms, color, PPO, and POD activities change in button mushrooms stored for 15 days at 5%, least significant difference.

Source	Dependent variable	*df*	Mean square	*F*	Significance
Corrected model	Firmness	19	0.167	426.450	0.000
	Weight loss	19	1.667	2.978E3	0.000
	Percent open caps	19	2349.934	1.319E6	0.000
	Marketability	19	5.028	4.055E3	0.000
	Total phenolics	19	9006.952	3.727E6	0.000
	Ascorbic acid	19	126.834	7.092E4	0.000
	Microbiological changes	19	3.904	5.841E3	0.000
	Color *L*	19	186.785	2.606E5	0.000
	Color Δ*E*	19	165.690	6.340E4	0.000
	Color BI	19	347.637	2.620E5	0.000
	PPO activity	19	443.120	6.438E5	0.000
	POD activity	19	226.072	1.840E5	0.000
Intercept	Firmness	1	15,875.941	4.053E7	0.000
	Weight loss	1	43.078	7.693E4	0.000
	Percent open caps	1	56,952.901	3.197E7	0.000
	Marketability	1	606.108	4.888E5	0.000
	Total phenolics	1	2.883E7	1.193E10	0.000
	Ascorbic acid	1	54,049.211	3.022E7	0.000
	Microbiological changes	1	1559.376	2.333E6	0.000
	Color *L*	1	413,185.634	5.765E8	0.000
	Color Δ*E*	1	39,803.322	1.523E7	0.000
	Color BI	1	56,878.367	4.287E7	0.000
	PPO activity	1	77,811.128	1.130E8	0.000
	POD activity	1	27,512.707	2.240E7	0.000
Treatments	Firmness	4	0.207	528.462	0.000
	Weight loss	4	0.376	671.618	0.000
	Percent open caps	4	94.543	5.306E4	0.000
	Marketability	4	0.397	320.114	0.000
	Total phenolics	4	5577.551	2.308E6	0.000
	Ascorbic acid	4	45.652	2.553E4	0.000
	Microbiological changes	4	0.948	1.418E3	0.000
	Color *L*	4	19.345	2.699E4	0.000
	Color Δ*E*	4	13.367	5.115E3	0.000
	Color BI	4	28.840	2.174E4	0.000
	PPO activity	4	110.944	1.612E5	0.000
	POD activity	4	38.596	3.142E4	0.000
Time	Firmness	3	0.631	1.612E3	0.000
	Weight loss	3	9.758	1.743E4	0.000
	Percent open caps	3	14,609.406	8.200E6	0.000
	Marketability	3	30.960	2.497E4	0.000
	Total phenolics	3	46,147.500	1.910E7	0.000
	Ascorbic acid	3	708.331	3.961E5	0.000
	Microbiological changes	3	22.701	3.397E4	0.000
	Color *L*	3	1138.015	1.588E6	0.000
	Color Δ*E*	3	1017.404	3.893E5	0.000
	Color BI	3	2135.153	1.609E6	0.000
	PPO activity	3	2594.358	3.769E6	0.000
	POD activity	3	1354.021	1.102E6	0.000
Treatments × time	Firmness	12	0.038	96.161	0.000
	Weight loss	12	0.075	134.358	0.000
	Percent open caps	12	36.863	2.069E4	0.000
	Marketability	12	0.088	71.036	0.000
	Total phenolics	12	864.949	3.579E5	0.000
	Ascorbic acid	12	8.521	4.765E3	0.000
	Microbiological changes	12	0.189	283.401	0.000
	Color *L*	12	4.791	6.685E3	0.000
	Color Δ*E*	12	3.536	1.353E3	0.000
	Color BI	12	7.024	5.295E3	0.000
	PPO activity	12	16.036	2.330E4	0.000
	POD activity	12	6.577	5.355E3	0.000

PPO, polyphenoloxidase; POD, peroxidase; BI, Browning index.

### Weight loss

Weight loss of button mushrooms increased during storage (Table 2). Weight of the mushrooms treated with unencapsulated and microencapsulated oils decreased, but compared to controls weight loss of microencapsulated treated was less. After 15 days of storage the controls had the most weight loss. The vapor-phase diffusion driven by a gradient of water vapor pressure at different locations was the reason for moisture loss. A reduction in weight loss was with treatment of encapsulated and microencapsulated oils compared to controls and delayed mushroom shriveling and quality deterioration, possibly due to decreased respiration rates. Experiments using eugenol, thymol, or menthol gases produced benefits due to reduced weight loss (Martinez-Romero et al. 2005; Serrano et al. 2005).

**Table 2:** Effect of unencapsulated and microencapsulated thyme and rosemary oils on firmness, weight loss, percent open caps, marketability, total phenolics, ascorbic acid, total aerobic plate count of microorganisms, and color change in button mushrooms stored at 4 ± 0.5°C for 15 days.

Quality parameter	Storage period (days)	Treatments				
		Control	TO	RO	MTO	MRO
Firmness (*N*)	0	16.51a	16.51a	16.51a	16.51a	16.51a
	5	16.17d	16.23c	16.32b	16.40a	16.44a
	10	15.98c	16.21b	16.16b	16.32a	16.30a
	15	15.59d	16.02c	16.09b	16.23a	16.20a
Weight loss (%)	0	0	0	0	0	0
	5	0.61d	0.50c	0.42b	0.41b	0.34a
	10	1.51d	1.03c	0.91b	0.85a	0.84a
	15	2.42d	1.72b	1.84c	1.73b	1.60a
Percent open caps (%)	0	0	0	0	0	0
	5	13.51d	12.22b	12.31c	12.15b	11.95a
	10	45.69e	41.35d	39.64c	39.09b	38.19a
	15	81.99e	73.36d	66.22c	65.21b	63.21a
Marketability	0	5	5	5	5	5
	5	3.31d	3.52c	3.51c	3.65b	3.75a
	10	2.01d	2.15c	2.24b	2.89a	2.90a
	15	1.39e	1.66d	1.72c	1.81b	1.91a
Total phenolics (mg·kg^−1^)	0	756.12a	756.12a	756.12a	756.12a	756.12a
	5	680.53b	710.11a	722.43a	730.02a	741.51a
	10	611.34b	672.53a	681.19a	692.14a	699.13a
	15	580.21b	642.76a	633.11a	621.64a	663.29a
Ascorbic acid (mg·kg^−1^)	0	38.32a	38.32a	38.32a	38.32a	38.32a
	5	30.33d	32.02c	33.14b	33.17b	35.16a
	10	21.56d	26.75b	24.23c	28.17a	28.24a
	15	18.21e	21.41d	23.21c	25.67b	27.14a
Microbiological changes (log CFU·g^−1^)	0	3.98a	3.98a	3.98a	3.98a	3.98a
	5	4.57c	4.11ab	4.13b	4.19b	4.08a
	10	6.32c	5.78b	5.75b	5.11a	5.13a
	15	7.22d	6.69c	6.74c	6.21b	6.11a
Color						
*L*	0	92.51a	92.31a	92.23a	92.57a	92.41a
	5	86.17d	88.23b	88.32b	87.40c	88.44a
	10	74.98c	78.21b	78.16b	81.32a	81.30a
	15	70.59e	73.02c	72.09d	74.23b	75.20a
Δ*E*	0	16.53ab	16.48a	16.42a	16.61b	16.50a
	5	22.17d	21.23b	21.32c	20.40a	20.44a
	10	33.98c	32.21b	32.16b	31.32a	31.30a
	15	37.59c	33.02b	32.09b	32.03b	31.20a
BI	0	19.51c	19.38b	18.32a	18.62a	19.50c
	5	23.17d	22.53c	22.49c	22.23a	22.35b
	10	41.98e	38.21d	37.16c	35.32b	34.30a
	15	48.59e	44.42d	43.29c	43.13b	41.21a

Data analyzed with least squares means, values in rows separated with least significant difference. BI, Browning index; MRO, microencapsulated rosemary oils; MTO, microencapsulated thyme oils. Means followed by the same letter in rows are not significantly different at *P* < 0.05.

### Percent open caps

Unencapsulated and microencapsulated oils delayed cap opening compared to controls at the end of storage (Table 2). Cap opening of mushrooms is related to dryness as a result of water loss during storage. Increased water loss during storage causes a decrease in cohesive forces of water and other hydrophilic molecules, such as proteins, responsible for the intact condition of mushroom caps and veils. The unencapsulated and microencapsulated oils reduced water loss; cap opening of mushrooms was less particularly for the microencapsulated rosemary oil (Gao et al. 2014).

### Marketability

Marketability was decreased in treated sporophores and control samples were unacceptable after 10 days of storage (Table 2). This might be due to high decay incidence and changes in carbohydrates, proteins, amino acids, and phenolic compounds that can influence color, texture, percent open caps, etc. Microcapsules with rosemary oil had the highest values after 15 days of storage; controls had the lowest. Only microencapsulated rosemary and thyme oils had acceptable marketable values at 10 days of storage.

### Total phenolic determination

Controls had the greatest decrease in total phenolics at 15 days of storage (Table 2). There was no difference in total phenolics between different essential oil treatments. Total free phenolic level in controls was less than that in essential oil treatments. Essential oils are a good source of antioxidant and antimutagenic phenolic (Kitazuru et al. 2004) that could be the reason of this treatment to reduce phenol changes.

### Determination of ascorbic acid

Total ascorbic acid contents of treated mushrooms during 15 days storage decreased (Table 2). Ascorbic acid maintenance was affected by the interaction of treatment and storage time. Use of all treatments reduced ascorbic acid loss. The microencapsulated rosemary oils were effective in retaining ascorbic acid levels. Essential oils could inhibit ascorbic acid loss, which may be due to the protection of antioxidant phenolics.

### Microbiological analysis

Total numbers of aerobic mesophilic microorganisms in control samples increased by the end of storage (Table 2). The treatments effectively inhibited growth of microorganisms; no differences between unencapsulated rosemary and thyme oils. The microencapsulated rosemary oil produced the most inhibition in growth of microorganisms. Jacxsens et al. (2000, 2002) reported that the critical limit for total aerobic plate count for vegetables is 10^8^ CFU·g^−1^, and that the microbial analysis in all treatments was less than this.

### Color

An important parameter in determining button mushroom acceptability by consumers is color. The mushroom color was modified as a result of storage, and both Δ*E* and BI were increased, but *L* value was reduced after 15 days of storage (Table 2). All treatments were able to maintain color quality. The *L* value of the microcapsule samples was acceptable at 10 days (Briones et al. 1992). After 15 days of storage, microencapsulated rosemary oils had the least change in *L*, Δ*E*, and BI values and were marketable. Antioxidant activity for these essential oils may reduce dehydration and occurrence of browned polymers.

### Activity of PPO and POD enzymes

**Figure 1:**
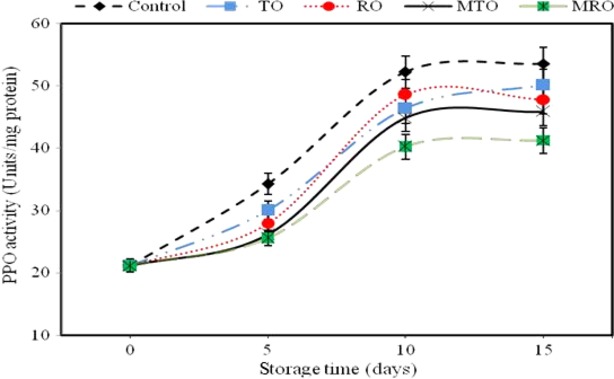
Effect of unencapsulated and microencapsulated thyme and rosemary oils on PPO 374 activity of button mushroom during 15 days of storage at 4 ± 0.5°C. PPO, polyphenoloxidase.

Control mushrooms showed a significant increase in PPO activity over time (Fig. 1). All treatments inhibited the increase in PPO activity. Microencapsulated-treated mushrooms had lowered PPO activity at day 15 storage (Fig. 1). Oxidation of phenolic substrates by PPO is believed to be a major cause of brown discoloration. The degree of browning in mushrooms was correlated with PPO activity and concentration of free phenolic substrates. The effect of low oxygen on browning is often attributed to PPO (Vámos-Vigyázó and Haard, 1981).

**Figure 2:**
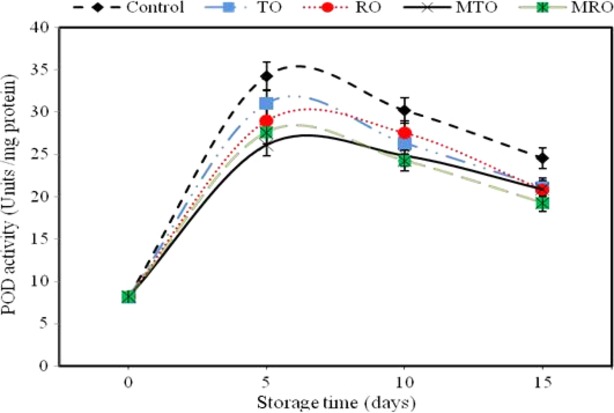
Effect of unencapsulated and microencapsulated thyme and rosemary oils on POD 379 activity of button mushroom during 15 days of storage at 4 ± 0.5°C. POD, peroxidase.

POD is an important oxyradical detoxification enzyme. This enzyme catalyzes more than one reaction and acts on a number of substrates, causing browning and leading to discoloration, off-flavors, and loss of nutrients. Inactivation of this enzyme is necessary to minimize deterioration. Increase in POD activities was observed during the first 4 days of storage in all treatments and decreased thereafter (Fig. 2). The POD activities in all treatments were lower than that in controls during storage time with difference occurring in all treatments. The POD activity in the microencapsulated rosemary oil treatment at the end of storage was lowest.

## Conclusions

Thyme and rosemary oils improved shelf-life of button mushroom. Methods used in preparation allowed essential oils to be entrapped without any change in their composition. Differences found in release patterns could be due to different hydrophilic characteristics of the oils. The high content of polar compounds in rosemary oil seems to favor entrapment of aqueous phase into microparticles during coacervation and provide a slower release. The greater content of small, polar compounds, present in the thyme oil, could favor a more rapid release. This may be due to different amounts of essential oil content in microcapsules of thyme and rosemary at the end of the test.

It remains to be determined if concentration of the essential oils or treatment timing could affect shelf-life differently. The encapsulation process is suitable for entrapping essential oils of different chemical composition. The method reduces loss of the active principles, leading to high-loaded microparticles that offer protection against environmental agents; it also offers the possibility of controlled release. Further experiments are needed to assess the suitability of natural active principle formulations for application as a new tool in integrated control of mushroom shelf-life.

## Conflict of Interest

None declared.
